# Whole-Genome Sequencing Reveals the Contribution of Long-Term Carriers in Staphylococcus aureus Outbreak Investigation

**DOI:** 10.1128/JCM.00363-17

**Published:** 2017-06-23

**Authors:** N. C. Gordon, B. Pichon, T. Golubchik, D. J. Wilson, J. Paul, D. S. Blanc, K. Cole, J. Collins, N. Cortes, M. Cubbon, F. K. Gould, P. J. Jenks, M. Llewelyn, J. Q. Nash, J. M. Orendi, K. Paranthaman, J. R. Price, L. Senn, H. L. Thomas, S. Wyllie, D. W. Crook, T. E. A. Peto, A. S. Walker, A. M. Kearns

**Affiliations:** a National Institute for Health Research Oxford Biomedical Research Centre, John Radcliffe Hospital, Oxford, United Kingdom; b Nuffield Department of Medicine, University of Oxford, Oxford, United Kingdom; c Antimicrobial Resistance and Healthcare Associated Infections Reference Unit, Public Health England, Colindale, United Kingdom; d Public Health England, Royal Sussex County Hospital, Brighton, United Kingdom; e Lausanne University Hospital, Service of Preventative Medicine, Lausanne, Switzerland; f Newcastle upon Tyne Hospitals NHS Foundation Trust, Newcastle, United Kingdom; g Portsmouth Hospitals NHS Trust, Portsmouth, United Kingdom; h Brighton and Sussex University Hospitals NHS Trust, Brighton, United Kingdom; i Plymouth Hospitals NHS Trust, Plymouth, United Kingdom; j Brighton and Sussex Medical School, Falmer, United Kingdom; k East Kent Hospitals NHS Foundation Trust, Canterbury, United Kingdom; l Royal Stoke University Hospital, University Hospitals of North Midlands NHS Trust, Stoke-on-Trent, United Kingdom; m Public Health England, London, United Kingdom; n National Institute for Health Research Health Protection Research Unit in Healthcare Associated Infections and Antimicrobial Resistance at University of Oxford, Oxford, United Kingdom; Cleveland Clinic

**Keywords:** MRSA, Staphylococcus aureus, outbreaks, whole-genome sequencing

## Abstract

Whole-genome sequencing (WGS) makes it possible to determine the relatedness of bacterial isolates at a high resolution, thereby helping to characterize outbreaks. However, for Staphylococcus aureus, the accumulation of within-host diversity during carriage might limit the interpretation of sequencing data. In this study, we hypothesized the converse, namely, that within-host diversity can in fact be exploited to reveal the involvement of long-term carriers (LTCs) in outbreaks. We analyzed WGS data from 20 historical outbreaks and applied phylogenetic methods to assess genetic relatedness and to estimate the time to most recent common ancestor (TMRCA). The findings were compared with the routine investigation results and epidemiological evidence. Outbreaks with epidemiological evidence for an LTC source had a mean estimated TMRCA (adjusted for outbreak duration) of 243 days (95% highest posterior density interval [HPD], 143 to 343 days) compared with 55 days (95% HPD, 28 to 81 days) for outbreaks lacking epidemiological evidence for an LTC (*P* = 0.004). A threshold of 156 days predicted LTC involvement with a sensitivity of 0.875 and a specificity of 1. We also found 6/20 outbreaks included isolates with differing antimicrobial susceptibility profiles; however, these had only modestly increased pairwise diversity (mean 17.5 single nucleotide variants [SNVs] [95% confidence interval {CI}, 17.3 to 17.8]) compared with isolates with identical antibiograms (12.7 SNVs [95% CI, 12.5 to 12.8]) (*P* < 0.0001). Additionally, for 2 outbreaks, WGS identified 1 or more isolates that were genetically distinct despite having the outbreak pulsed-field gel electrophoresis (PFGE) pulsotype. The duration-adjusted TMRCA allowed the involvement of LTCs in outbreaks to be identified and could be used to decide whether screening for long-term carriage (e.g., in health care workers) is warranted. Requiring identical antibiograms to trigger investigation could miss important contributors to outbreaks.

## INTRODUCTION

To manage Staphylococcus aureus outbreaks effectively, infection control practitioners need to determine the relatedness of isolates from suspected cases. Whole-genome sequencing (WGS) has shown superior resolution compared with standard typing techniques (*spa*, pulsed-field gel electrophoresis [PFGE]) when used for individual outbreaks (1–4) and can also provide additional information about resistance, pathogenicity, and population structure (5–8). However, it has been argued that the accumulation of within-host diversity during S. aureus carriage could result in erroneous inferences about transmission. This has been cited as a potential weakness in applying sequencing to S. aureus outbreaks and may lead to the misinterpretation of genuine transmission routes (1, 9, 10).

However, rather than within-host diversity being a limitation on sequencing-based outbreak investigation, it could in fact be exploited to determine whether a long-term carrier is implicated in maintaining an outbreak. This information could be used by infection control practitioners when considering whether or not to deploy extended screening (e.g., of health care workers).

In this study, we tested the hypothesis that WGS can be used to predict the presence of a long-term carrier as an outbreak source. First, we examined individuals with newly acquired S. aureus nasal carriage to ascertain whether diversity is present at acquisition or develops over time. Next, we analyzed 20 S. aureus outbreaks, which were previously investigated using standard typing techniques, to assess the added utility of WGS. Finally, we compared WGS with epidemiological data to determine whether the presence of a long-term carrier maintaining the outbreak could be inferred from the WGS data.

## RESULTS

### Comparison of within-host diversity in newly acquired and long-term carriage.

Eight subjects were identified with ≥3 consecutive bimonthly negative nasal swabs, followed by ≥1 year of swabs consistently positive for S. aureus. All isolates were methicillin-susceptible S. aureus (MSSA), representing 7 *spa* types, 5 sequence types, and 4 clonal complexes. The median time from the first to last positive sample was 490 days (range, 358 to 727 days). In total, 135 isolates were successfully sequenced from 16 samples. One isolate (case 1219, early sample) failed quality checks and was excluded.

**FIG 1:**
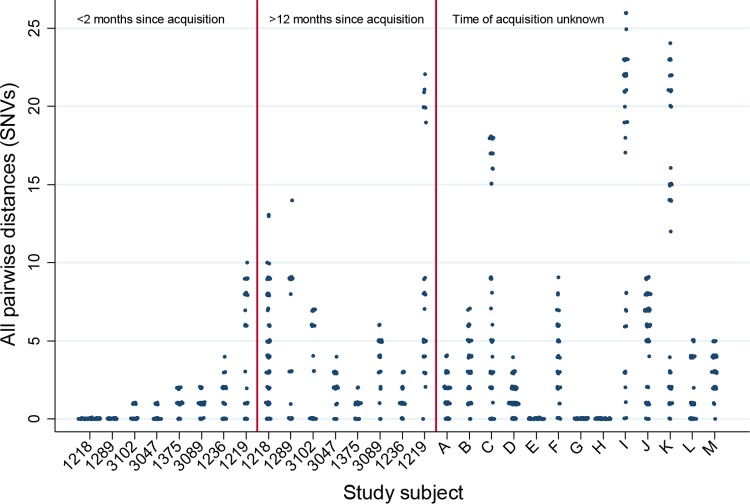
All pairwise differences between early (<2 months since acquisition) and late (>12 months since acquisition) nasal swab samples from 7 patients with previously negative nasal swabs. Included for comparison are samples from patients positive at entry to the study (time of acquisition unknown).

In 6/8 subjects, there was a significant increase in mean pairwise diversity (MPWD) between the first and last samples (*P* < 0.05) (Fig. 1). In one participant (case 1236), the increase was not significant (*P* = 0.52), and for another (case 1375), there was a decrease which was marginally significant (*P* = 0.07). Overall, MPWD increased from 0.88 single nucleotide variants (SNVs) (95% confidence interval [CI], 0.65 to 1.11) to 3.30 (95% CI, 2.92 to 3.68) between the first and last samples (*P* < 0.001). An analysis of the phylogenetic trees (see supplemental material) showed highly clonal early populations, and in 2 participants, only a single strain was observed. One individual (case 1219) had a more diverse early sample (MPWD, 4.57; 95% CI, 3.10 to 6.04) compared with those of the other participants. This subject's first positive swab was at month 12, and they had completed a course of amoxicillin-clavulanic acid 1 day before their final negative swab. Therefore, it is possible that this was a false negative due to antibiotic suppression, meaning that there may have been up to 4 months of carriage prior to the first positive swab, accounting for the increased diversity.

Two participants (cases 1218 and 1219) shared the same address and had isolates of the same *spa* type. Participant 1219 (donor) became positive 2 months before participant 1218 (recipient). On a direct comparison of both early populations, we found that the recipient had an entirely clonal initial population, identical to 4/8 of the donor's strains (see supplemental material).

For an additional 13 participants positive at the study entry, within-host diversity as measured by MPWD ranged from 0 SNVs (3 individuals) to 26 SNVs. This may be due to differences in the acquisition time to the time of the first sample, which is unknown for these individuals.

### Outbreak characteristics.

Twenty outbreaks were included in the study (Table 1). Fourteen (70%) were hospital associated, including 5 in neonatal units, 4 in general wards, 1 from a surgical unit, 2 from maternity units, and 2 involved multiple wards or hospital sites. Six (30%) were community associated, including 4 households, 1 nursing home, and 1 school. The reasons for instigating an outbreak investigation were an increase in methicillin-resistant S. aureus (MRSA) carriage (8 outbreaks), Panton-Valentine leukocidin (PVL)-producing skin/soft tissue infection (7 outbreaks), surgical site infections (3 outbreaks), MRSA bacteremia (1 outbreak), and staphylococcal scalded skin syndrome (1 outbreak). Three (15%) were due to MSSA and 17 (85%) to MRSA. The median number of outbreak cases was 7 (interquartile range [IQR], 5 to 9). The median duration was 72 days (IQR, 44 to 188 days).

**TABLE 1:** Description of 20 outbreaks analyzed by whole-genome sequencing

Outbreak	Epidemiological category	No of cases	Reason for outbreak investigation	MRSA or MSSA	Clonal complex	MLST^*a*^	*spa*	Duration (days)	PFGE pulsotypes	Outbreak antibiograms
A	Hospital, general ward	5	MRSA colonization	MRSA	CC22	ST22	t032	367	All identical	All identical
B	Hospital, general ward	6	S. aureus wound infections	MSSA	CC8	ST2021	t008	412	All identical	All identical
C	Hospital, general ward	7	S. aureus wound infections	MRSA	CC8	ST239	t037	98	All identical	All identical
D	Hospital, general ward	17	MRSA colonization	MRSA	CC8	ST8	t008	88	All identical	All identical
E	Hospital, surgical unit	8	S. aureus wound infections	MRSA	CC22	ST22	t022	18	All identical	All identical
F	Hospital, multiple wards	50	MRSA colonization	MRSA	CC5	ST228	t041	122	2 pulsotypes	All identical
G	Hospital, multiple wards	187	MRSA colonization	MRSA	CC8	ST8	t008	454	4 pulsotypes	3 antibiograms
H	Hospital, maternity unit	6	PVL-related SSTIs^*b*^	MRSA	CC1	ST772	t657	70	All identical	All identical
I	Hospital, maternity unit	9	Scalded skin syndrome	MSSA	CC15	ST2434	t346	70	All identical	2 antibiograms
J	Hospital, neonatal unit	3	MRSA colonization	MRSA	CC59	ST59	t216	8	All identical	All identical
K	Hospital, neonatal unit	4	MRSA colonization	MRSA	CC22	ST22	t5892	43	All identical	All identical
L	Hospital, neonatal unit	6	MRSA colonization	MRSA	CC30	ST30	t019	57	All identical	All identical
M	Hospital, neonatal unit	8	MRSA bacteremia	MRSA	CC88	ST88	t5973	65	All identical	3 antibiograms
N	Hospital, neonatal unit	41	MRSA colonization	MRSA	CC22	ST22	t5892	1526	All identical	2 antibiograms
O	Household	3	PVL-related SSTIs	MRSA	CC30	ST30	t019	8	All identical	All identical
P	Household	4	PVL-related SSTIs	MRSA	CC30	ST30	t019	20	3 pulsotypes	All identical
Q	Household	5	PVL-related SSTIs	MRSA	CC30	ST30	t019	195	2 pulsotype	All identical
R	Household	8	PVL-related SSTIs	MRSA	CC30	ST30	t019	44	All identical	2 antibiograms
S	Nursing home	9	PVL-related SSTIs	MRSA	CC30	ST30	t019	298	2 pulsotypes	3 antibiograms
T	School	5	PVL-related SSTIs	MSSA	CC121	ST121	t645	74	All identical	All identical

a MLST: multi-locus sequence-type.

b PVL: Panton-Valentine leukocidin; SSTI, skin/soft tissue infection.

Overall, isolates from 391 cases were sequenced. Nine (2.3%) were from health care workers (HCWs), the remainder being from patients or household members. Outbreak samples represented 9 clonal complexes, 11 sequence types, and 12 *spa* types.

### Phylogenetic analysis of outbreaks.

Phylogenetic trees for each outbreak are provided in the supplemental material. Two outbreaks had isolates which were equally or more distant than comparator isolates despite having the outbreak pulsotype: outbreak D (one isolate, 53 SNVs from the index case compared with 21) and outbreak S (two isolates, 49 and 46 SNVs from the index case compared with 46). These were therefore considered to be sporadic nonoutbreak isolates and were excluded from further analysis.

The overall MPWD across all outbreak sample pairs for the remaining 388 isolates was 13.8 SNVs (95% CI, 13.6 to 13.9) compared with 4,444 SNVs for nonoutbreak *spa*-matched pairs (95% CI, 2,492 to 6,395) and 30,192 SNVs for nonoutbreak isolates from the same units (95% CI, 29,781 to 30,603). All outbreak isolates were ≤30 SNVs from the index case; 381/388 (98%) were ≤10 SNVs from their nearest neighbor. The 7 more distant isolates came from outbreaks lasting more than 6 months (B, G, and S). All isolates were mapped to a standard reference genome; mapping to an alternative reference strain (performed for 6 outbreaks) yielded only 2 additional SNVs overall (see supplemental material), with no effect on topology.

### Time to most recent common ancestor and long-term carriers.

**FIG 2:**
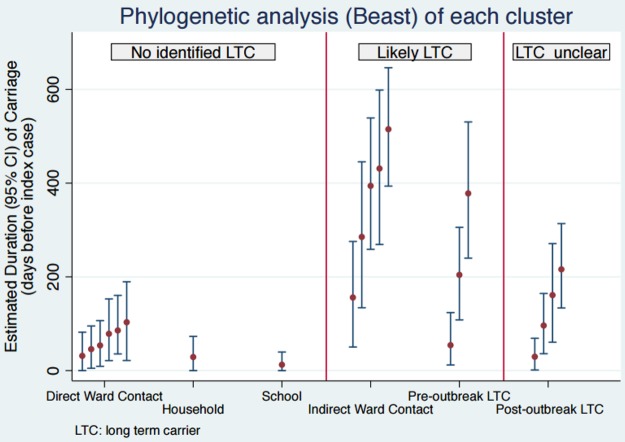
Duration-adjusted TMRCA for outbreaks with (i) no evidence of a long-term carrier (direct contacts between all cases); (ii) likely LTC (indirect ward contacts or preoutbreak LTC); or (iii) LTC unclear/possible (evidence of a postoutbreak LTC).

Twelve outbreaks (60%) had epidemiological evidence of a long-term carrier (LTC). Three included cases with recurrent staphylococcal disease, in 5 an LTC was suspected due to nonoverlapping ward stays, and in 4, at least one case had postoutbreak long-term carriage (Fig. 2). The pairwise distances between isolates from outbreaks with evidence for an LTC ranged from 0 to 46 SNVs compared with 0 to 10 SNVs for outbreaks with no evidence for an LTC (Table 2). The mean duration-adjusted time to most recent common ancestor (TMRCA) for outbreaks with a suspected or proven LTC was 243 days (95% highest posterior density interval [HPD], 143 to 343 days) compared with 55 days (95% HPD, 28 to 81 days) for outbreaks with no evidence for an LTC (*P* = 0.004) (Fig. 2). Excluding postoutbreak carriage, an analysis of the receiver operating characteristic curve gave an area under the curve (AUC) of 0.953 (95% CI, 0.851 to 1). Using the Youden index to select the optimal threshold gave a cutoff value of 156 days, with a sensitivity of 0.875 and a specificity of 1.

**TABLE 2:** Long-term carrier category, duration-adjusted TMRCA, and SNV range for outbreaks investigated using WGS

Outbreak	Long-term carrier category	Duration-adjusted TMRCA (days [95% highest posterior density interval])	Distance between all isolates in cluster (SNV range)
A	Indirect ward contact	285 (134–445)	0–19
B	Indirect ward contact	515 (394–646)	0–24
C	Direct ward contact	103 (61–228)	0–9
D	Direct ward contact	78 (21–153)	0–10
E	Postoutbreak LTC	96 (40–165)	0–6
F	Direct ward contact	86 (36–160)	0–5
G	Indirect ward contact	394 (259–539)	0–46
H	Direct ward contact	46 (5–95)	0–4
I	Postoutbreak LTC	30 (1–69)	0–5
J	Postoutbreak LTC	161 (61–271)	0–9
K	Direct ward contact	31 (0–82)	1–4
L	Postoutbreak LTC	216 (134–314)	0–8
M	Direct ward contact	54 (9–107)	0–4
N	Indirect ward contact	156 (50–275)	0–36
O	Preoutbreak LTC	378 (240–531)	9–25
P	Preoutbreak LTC	54 (12–124)	1–11
Q	Preoutbreak LTC	204 (108–306)	1–13
R	Household	29 (0–73)	1–2
S	Indirect ward contact	431 (269–599)	3–32
T	School	12 (0–40)	0–2

### Relationship between PFGE pulsotype/antibiogram and SNV distance.

Five outbreaks contained isolates differing by ≥1 band from the index case on PFGE. MPWD between outbreak isolates with identical PFGE pulsotypes was 13.6 SNVs (95% CI, 13.4 to 13.7) compared with 17.3 (95% CI, 17.0 to 17.6) between isolates with differing pulsotypes (*P* < 0.0001).

**FIG 3:**
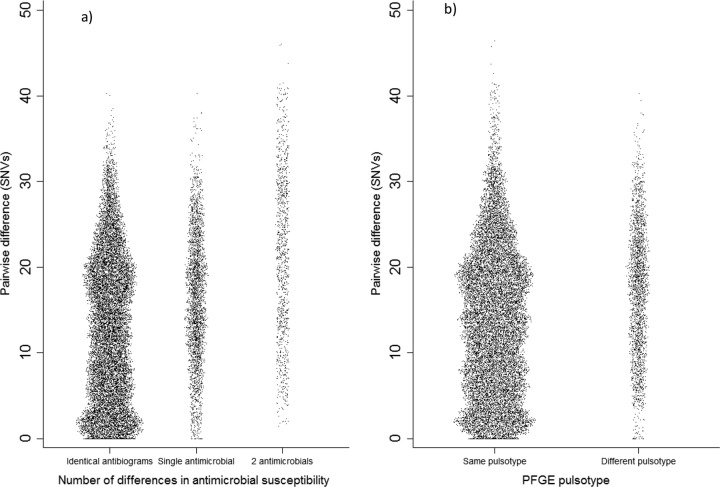
Pairwise SNV differences for all pairs within an outbreak, where isolates had differing antibiograms (a) or differing PFGE pulsotypes (b).

In 6/20 outbreaks, antimicrobial susceptibility differed across isolates, confirmed by the presence/absence of mobile resistance determinants identified using BLAST (11); however, these clearly belonged to the outbreak on phylogenetic analysis. MPWD between isolates sharing an antibiogram was 12.7 SNVs (95% CI, 12.5 to 12.8) compared with 17.5 (95% CI, 17.3 to 17.8) for isolates with differing antibiograms (*P* < 0.0001), although a substantial number of isolate pairs with different antibiograms had 0 SNVs between their core genomes (Fig. 3).

For other factors potentially related to outbreak diversity, there was no evidence of an association between MPWD and outbreak duration, reason for investigation, epidemiological setting, or MRSA phenotype (*P* > 0.05).

## DISCUSSION

We have tested the use of WGS for S. aureus outbreak investigation using 20 outbreaks. By comparing observed outbreak SNV distances with nonoutbreak and *spa*/multilocus sequence type (MLST)-specific diversities, we were able to distinguish outbreak from nonoutbreak strains.

Our observation of minimal diversity in recent acquisitions of nasal carriage is reassuring for the application of WGS data to outbreaks. For the donor-recipient pair, we observed a narrow transmission bottleneck, with a clonal founding population despite a diverse donor population. Although this is a single case, the findings are supported by the minimal diversity seen in the early samples for the majority of carriage study subjects, and further evidence for a narrow transmission bottleneck is provided by the relatively short SNV distances observed across the outbreaks. Taken together, these findings suggest that, in an acute short-term outbreak, there will be insufficient time for diversity to accumulate.

If WGS is to be used routinely for outbreak investigation, these findings provide evidence that single-colony sequencing is likely to identify clusters reliably in this context, allowing for the ease of interpretation and ensuring that WGS remains an affordable alternative to standard typing, as a requirement for sequencing multiple colonies per case, as implied by previous investigators (1, 10), would rapidly escalate costs and render WGS too expensive for routine use.

Previous carriage studies have found greater distances than seen here (9, 11); however, these did not account for the estimated time of acquisition. We postulate that the existence of a significant cloud of diversity (4, 12) may be a marker of long-term carriage. Therefore, in outbreaks, higher diversity may indicate the involvement of an LTC, with outbreak diversity reflecting the donor cloud.

In support of this, we observed a significant difference in duration-adjusted TMRCAs between outbreaks with and without evidence of an LTC. The longest TMRCAs were in hospital outbreaks with indirect links between cases (i.e., nonoverlapping ward stays). The likelihood of “missed” cases in these outbreaks was considered low due to the enhanced screening, and the most likely reason for the reoccurrence of the outbreak strain was thought by the investigating teams to be either a reintroduction from the community (outbreak G) or from a staff member with long-term carriage (outbreaks A, I, N, and S). Staff carriage was proven in one outbreak (by sampling and subsequent termination of the outbreak on their exclusion), but in the remaining outbreaks, HCWs were either not sampled or HCW sampling was anonymized and positive results could not be linked definitively with the suspected carrier.

The outbreaks included in this study necessarily reflect the circulating S. aureus clones in the United Kingdom and the concerns of local infection control teams. The sampling frame is therefore enriched for MRSA and PVL-positive outbreaks and those from neonatal units. Despite this, there is a wide representation of sequence types.

In spite of the enhanced surveillance during each outbreak, there inevitably are missing transmission links, due to missed sampling, suppression from antimicrobial therapy, or delays in identifying contacts. One reason for missed samples may be the use of antibiograms as an initial screening tool for identifying putative outbreak isolates, as most investigating teams only collected isolates with identical or highly similar antimicrobial susceptibility profiles. However, in the six outbreaks where isolates were included with differing antibiograms, the core genomes were remarkably conserved. This is presumably due to the ready loss/gain of mobile genetic elements (13) and shows that reliance on antibiograms may lead to samples being wrongly excluded.

The variability of mobile elements is also important for interpreting genetic distances. Recombination events such as the gain/loss of a mobile element will introduce a large number of SNVs even though this represents a single genetic event. Current analysis tools which can accommodate this are computationally complex and, for large data sets, require sizable computing resources. A simpler approach is to exclude the “mobile-ome” from phylogenetic analyses and compare only the core genome, and the results above demonstrate that this is an acceptable strategy. Similarly, mapping to alternative reference strains (performed for six outbreaks) had minimal effects on SNV analysis and phylogeny, removing the need for identification of clonal complex or assembly of index case prior to phylogenetic analysis. This streamlined approach brings WGS closer to routine use, as a readily deployable method with a minimal burden of computational time and bioinformatics expertise.

In conclusion, we have demonstrated how a WGS-based approach can be applied to S. aureus outbreak investigations. We have shown that current sampling strategies provide sufficient information to determine whether isolates belong to an outbreak, and that, rather than confounding the investigation, within-host diversity can be utilized to identify the possible involvement of a long-term carrier, potentially enhancing the infection control response. Combining this with directed multisampling of suspected LTCs (1) may be a cost-effective method of using WGS to ensure that, where HCWs are implicated, potentially career-altering decisions are made using the best possible evidence.

## MATERIALS AND METHODS

### Comparison of within-host diversity in newly acquired and long-term carriage.

Eight participants were identified from a population study of S. aureus nasal carriage in adults attending general practices in Oxfordshire (15), in which participants had nasal swabs taken at two-month intervals, with positive samples stored as mixed glycerol stocks taken by sweeping across multiple colonies on the primary plates to preserve the diversity of carried strains (11). The eight participants were negative for nasal carriage at recruitment and subsequently had consistently negative swabs for ≥6 months before acquiring a strain which they carried for at least 1 year. The first and last positive samples for each individual were retrieved from the mixed glycerol stocks. Samples were plated on Columbia blood agar (CBA) and incubated overnight at 37°C. For each time point, 8 individual colonies (12 for case no. 1218) were selected and further subcultured on a CBA plate and again incubated overnight at 37°C.

We also retrieved sequencing data from 13 participants previously investigated, (9) for whom the approximate time of acquisition was unknown. Each of these had 8 to 12 individual colonies sequenced.

### Collection of outbreak isolates and epidemiological data.

Nineteen outbreaks were purposively sampled in collaboration with the Public Health England (PHE) staphylococcal reference laboratory, representing a range of sequence types and epidemiological settings and including both MRSA and MSSA. One further outbreak was investigated in conjunction with Lausanne University Hospital, Switzerland (14, 16). Epidemiological information was obtained from each infection control team (specimen date, site, ward location, and where applicable, admission/discharge dates and previous screening results).

For each outbreak, additional background isolates were also included for comparison. We sequenced all isolates submitted to PHE as part of the outbreak investigation, including those identified as “nonoutbreak” by routine typing, to estimate the expected genetic diversity of the outbreak strain and to ensure that the apparent outbreak strains were not part of an ongoing clonal expansion. We also included non-epidemiologically linked isolates matched for *spa* type and/or MLST to provide a comparison for expected within-*spa* distances and to provide an outgroup for phylogenetic analysis.

Isolates were retrieved from single-colony frozen stocks held at the PHE reference laboratory, Colindale, London, or at Lausanne University Hospital. We used only the first isolate from each case and included isolates both from clinical samples and screening swabs.

### Extraction and sequencing.

DNA was extracted and sequenced as previously described (6) from a single colony subcultured on CBA and incubated for 18 to 24 h. Sequencing was performed using the Illumina HiSeq or MiSeq platforms.

### Genome assembly and construction of phylogenetic trees.

For all outbreaks, reads were aligned using Stampy v1.0.17 to a standard reference genome (MRSA252; GenBank no. NC_002952) (17). Six outbreaks were also mapped to clonal complex-specific reference genomes obtained from in-house collections or GenBank. Single nucleotide variants were identified across all mapped nonrepetitive sites using SAMtools v 0.1.18 mpileup, with the extended base-alignment quality flag and masking of mobile genetic elements. A consensus of ≥75% and ≥5 reads, including one in each direction, were required to support an SNV, and calls were required to be homozygous under a diploid model. Maximum likelihood trees were estimated from the mapped whole genomes using PhyML (18).

### Outbreak analysis and calculation of TMRCA.

The index case was defined as the earliest microbiologically confirmed case in each cluster. Outbreak cases were defined as those sharing related PFGE pulsotypes (19) plus a definite epidemiological link to the index or secondary cases (>24-h stay in the same ward or household/classroom/similar community situation with prolonged contact, e.g., childcare). For each outbreak case, the genetic distance in SNVs was calculated from the index case and the nearest neighbor. If an isolate was more distant from the index case than the nearest *spa*/MLST-matched comparator, it was considered sporadic and excluded from further outbreak analysis.

We classified each outbreak according to the possibility of long-term carrier involvement (LTC carrying for ≥6 weeks) as follows: (i) LTC not suspected, direct contact between cases, or no history of preexisting staphylococcal disease; (ii) evidence for a pre- or perioutbreak LTC, either ≥1 case with a history of recurrent staphylococcal disease or nonoverlapping hospital stays (ward case identified after a case-free interval, indicating a possible health care worker carrier); and (iii) evidence of a postoutbreak LTC, ≥1 case with positive nasal swab >6 weeks after initial swab (indicating a propensity for long-term carriage).

To evaluate the relationship between outbreak diversity and the likelihood of a long-term carrier, we estimated the time to most recent common ancestor (TMRCA) using BEAST v1.8.1 (20). We applied a simple HKY substitution model with constant population size and a standardized substitution rate of 3.3 × 10^−6^ substitutions per genome per year (7) (see supplemental material). To control for differences in outbreak duration, outbreaks were censored at 6 months, and the (censored) outbreak duration was subtracted from the calculated TMRCA to obtain a duration-adjusted TMRCA.

We compared SNV distances between isolates of identical pulsotypes and those differing by one or more band. To determine whether there was an increase in genetic diversity associated with the acquisition of antimicrobial resistance, we also interrogated the predicted antibiograms as previously described (21).

Statistical analyses were performed using Stata v13.1. Mean pairwise differences were modeled using normal linear regression using robust standard errors to account for dependence within person/outbreak. The ability of TMRCA to differentiate between outbreaks with evidence for an LTC compared with outbreaks with no evidence for an LTC was evaluated using a receiver operating characteristic curve analysis.

### Accession number(s).

The sequences reported in this paper have been deposited in the NCBI Sequence Read Archive under bioproject number PRJNA380544.

## Supplementary Material

Supplemental material
