# Supplementary material for: Whole-Genome Sequencing Reveals the Contribution of Long-Term Carriers in Staphylococcus aureus Outbreak Investigation
Source: J Clin Microbiol. 2017 Jun 23;55(7):2188–97. doi: 10.1128/JCM.00363-17 (PMC5483921; doi:10.1128/JCM.00363-17)
Supplement: Supplemental material [file supp_55_7_2188__index.html]

Supplemental material 

# Whole-Genome Sequencing Reveals the Contribution of Long-Term Carriers in Staphylococcus aureus Outbreak Investigation

## Supplemental material

- Supplemental file 1 -

  Supplemental methods; Fig. S1 (Unrooted phylogenetic trees showing single colony picks from early and late samples from 8 subjects with ≥3 consecutive negative nasal swabs followed by ≥1 year of consistently positive swabs with closely related *spa* types), S2 (Timeline showing acquisition of MSSA, *spa* type t012, by 2 members of the same household), S3 (Unrooted PhyML tree showing first and last samples from household subjects A and B), and S4 (Phylogenetic trees for 20 outbreaks investigated using WGS); and Table S1 (Additional SNVs identified by mapping to alternative reference genomes for 6 outbreaks investigated by WGS)

  PDF, 1.0M
