# Supplementary material for: Whole-Genome Sequencing Reveals the Contribution of Long-Term Carriers in Staphylococcus aureus Outbreak Investigation
Source: J Clin Microbiol. 2017 Jun 23;55(7):2188–97. doi: 10.1128/JCM.00363-17 (PMC5483921; doi:10.1128/JCM.00363-17)
Supplement: Supplemental material [file JCM.00363-17_zjm999095560s1.pdf]

Supplementary data

1. Beast Analysis

BEAST analysis was performed using a simple HKY substitution model with a coalescent model and constant population size. The substitution rate was standardized for all outbreaks at a rate of 3.3×10^{-6} per genome per year.[1] Priors were set as follows: nucleotide frequencies: uniform prior; κ: log-normal prior with mean 1 and SD 1.25 on a logarithmic scale. Operators were set to auto-optomise. MCMC chain length was set to 10,000,000 with sampling every 1000 iterations and a burn in of 100,000 iterations, to obtain an effective sample size (ESS) of >200 for each iteration. Each sample was run in duplicate and the chains merged to obtain the final results.

1.Harris SR, Feil EJ, Holden MT, et al. Evolution of MRSA during hospital transmission and intercontinental spread. *Science* 2010; **327**(5964): 469-74.

2. Within host diversity.

Figure S1: Unrooted phylogenetic trees showing single colony picks from early (light blue) and late (dark blue) samples from 8 subjects with ≥3 consecutive negative nasal swabs followed by ≥1 year of consistently positive swabs with closely related *spa*-types. M12(1): month 12, colony 1.

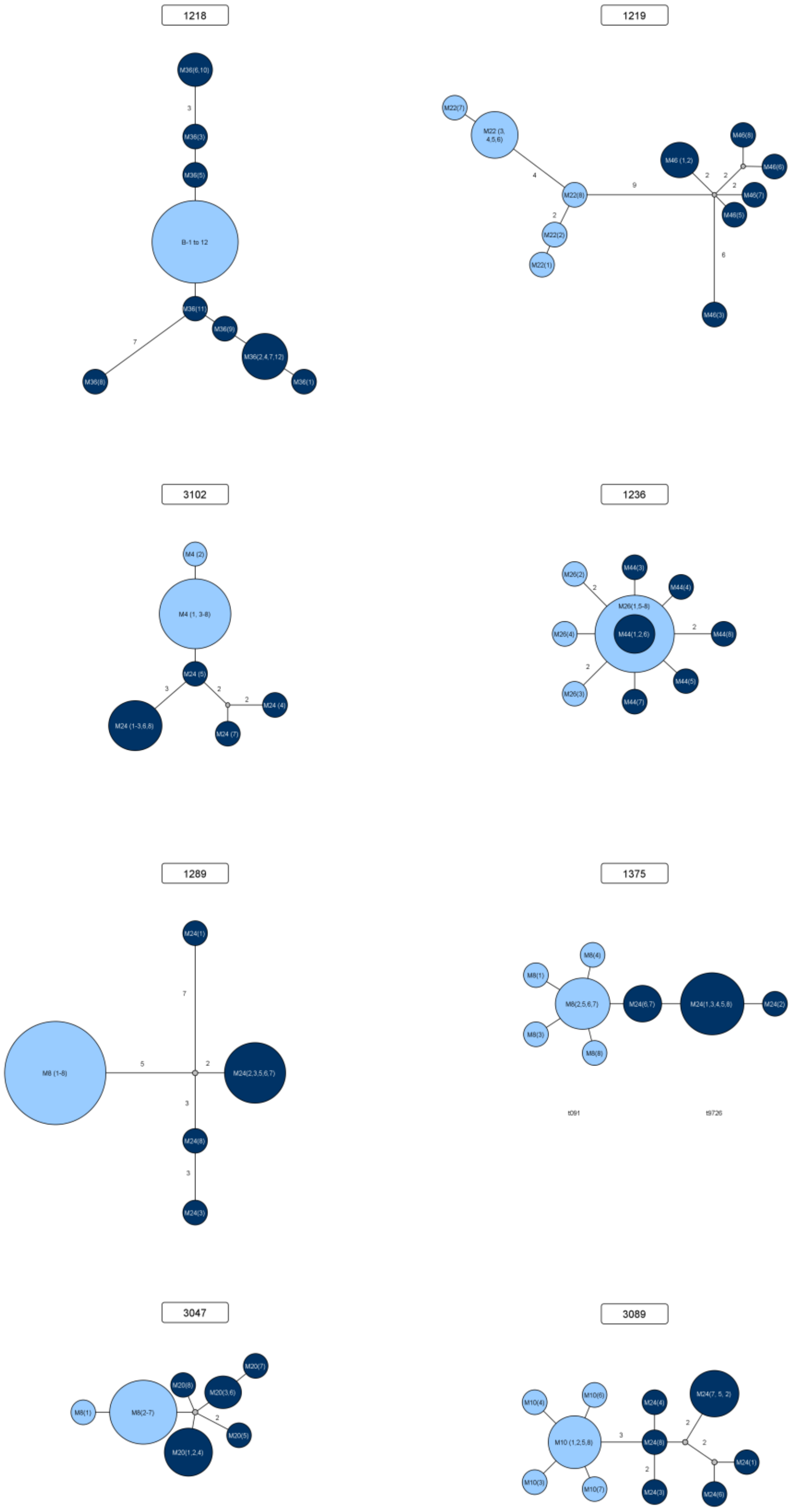

3. Comparison of nasal populations from household contacts.

Two subjects, 1218 (hereafter subject B) and 1219 (hereafter subject A), shared the same surname and address and were thus presumed to be household contacts. Subject A had 10 negative swabs before receiving a course of co-amoxiclav in month 19. The last antibiotic dose was taken the day before the 11th swab, which was negative. However, the 12th swab 2 months later was positive for MSSA (*spa* type t012) and the participant continued to return positive swabs, all of which were *spa* type t012, for a further 2 years. Subject B had 13 negative swabs and became positive 2 months after 1219, also with *spa* type t012 which was consistently carried for 12 months (figure S2).

Figure S2: timeline showing acquisition of MSSA, *spa* type t012 by 2 members of the same household (subject A (1218), subject B (1219)).

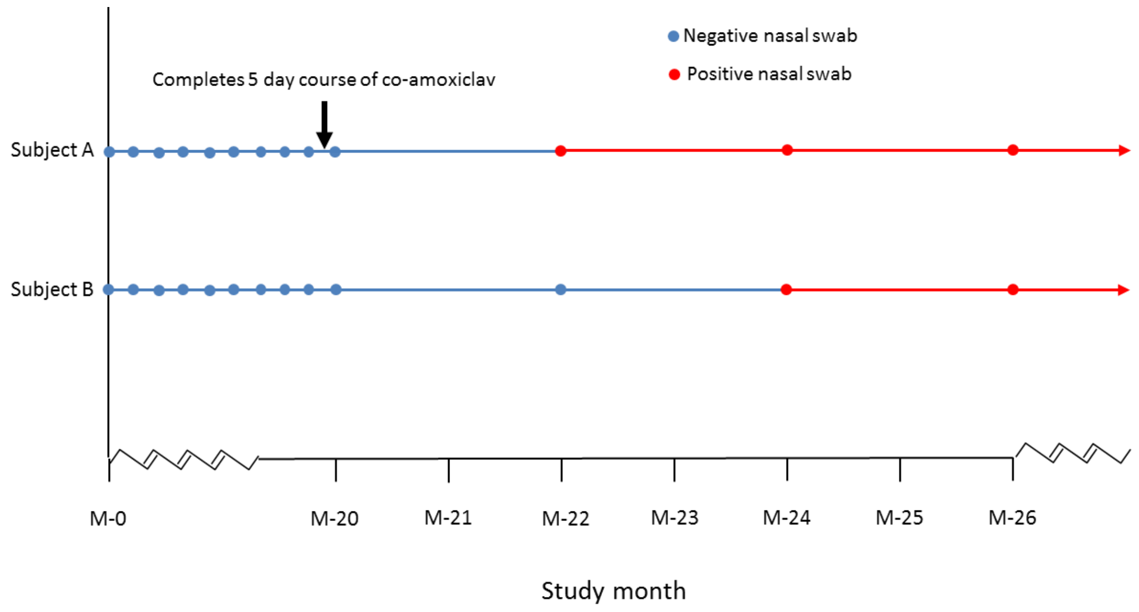

Figure S3: Unrooted PhyML tree showing first and last samples from household subjects A and B. Light blue circles: subject A, early; dark blue circles: subject A, late; light pink: subject B, early; dark pink: subject B, late. Branch lengths are given in SNVs. Numbers in circles indicate colony pick number.

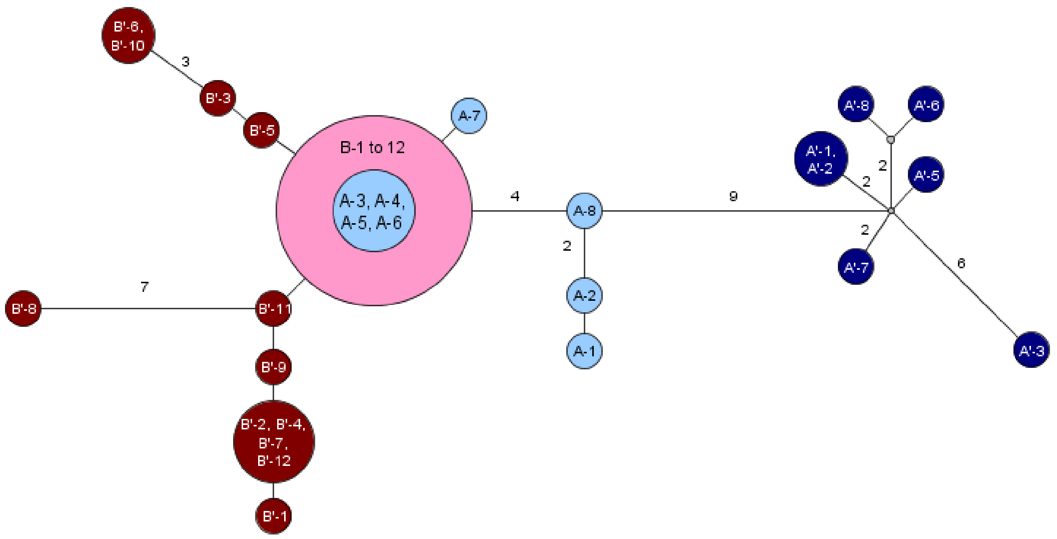

4. Phylogenies of outbreaks investigated by WGS

Figure S4. Phylogenetic trees for twenty outbreaks investigated using WGS. Branch lengths are labelled in SNV distance (if greater than 2). If there is an interval of > 6 months between subclusters within an outbreak, later samples are shaded dark grey.

Nodes are labelled chronologically. The nearest, non-epidemiologically linked *spa* or MLST matched comparator isolates (one included per outbreak) are indicated by an empty circle. Octagonal nodes (outbreaks D and S) indicate putative outbreak isolates which, on analysis, were as or more distant to the index case than the nearest non-linked comparator isolate, and therefore considered presumed sporadic isolates.

P1: first case in outbreak. HCW: healthcare worker. W1: week 1 of outbreak.

◊ indicates a mobile element encoding resistance not present in all outbreak isolates.

Dashed lines indicate a contracted branch, and a thick line indicates a branch which has been elongated to allow visualisation.

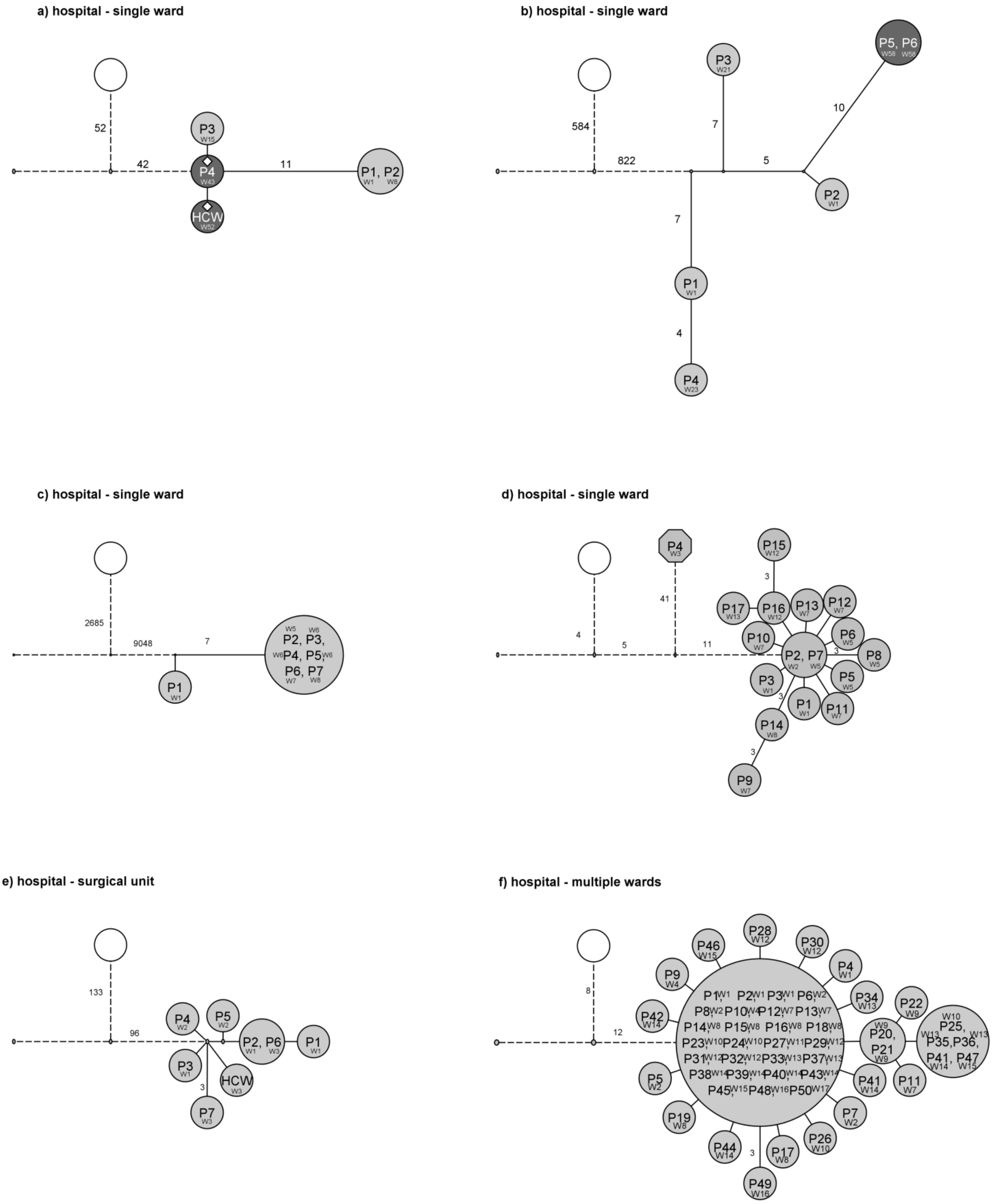
a) hospital - single ward
52
42
11
P3
W15
P4
W43
P1, P2
W1 W8
HCW
W52
b) hospital - single ward
584
822
7
5
10
7
4
P3
W21
P5, P6
W58 W58
P2
W1
P1
W1
P4
W23
c) hospital - single ward
2685
9048
7
P1
W1
P2, P3,
P4, P5,
P6, P7
d) hospital - single ward
P4
W3
41
4
5
11
P15
W12
P17
W13
P16
W12
P13
P12
P10
P6
P2, P7
P8
P3
P5
P1
P11
P14
P9
e) hospital - surgical unit
133
96
3
P4
W2
P5
W2
P2, P6
W1 W3
P1
W1
P3
W1
HCW
W3
P7
W3
f) hospital - multiple wards
8
12
3
P28
W12
P30
W12
P46
W15
P9
W4
P4
W1
P34
W13
P22
W9
P42
W14
P20,
P21
P25,
P35,P36,
P41, P47
P5
W2
P41
W14
P11
W7
P19
W8
P7
W2
P26
W10
P44
W14
P17
W8
P49
W16

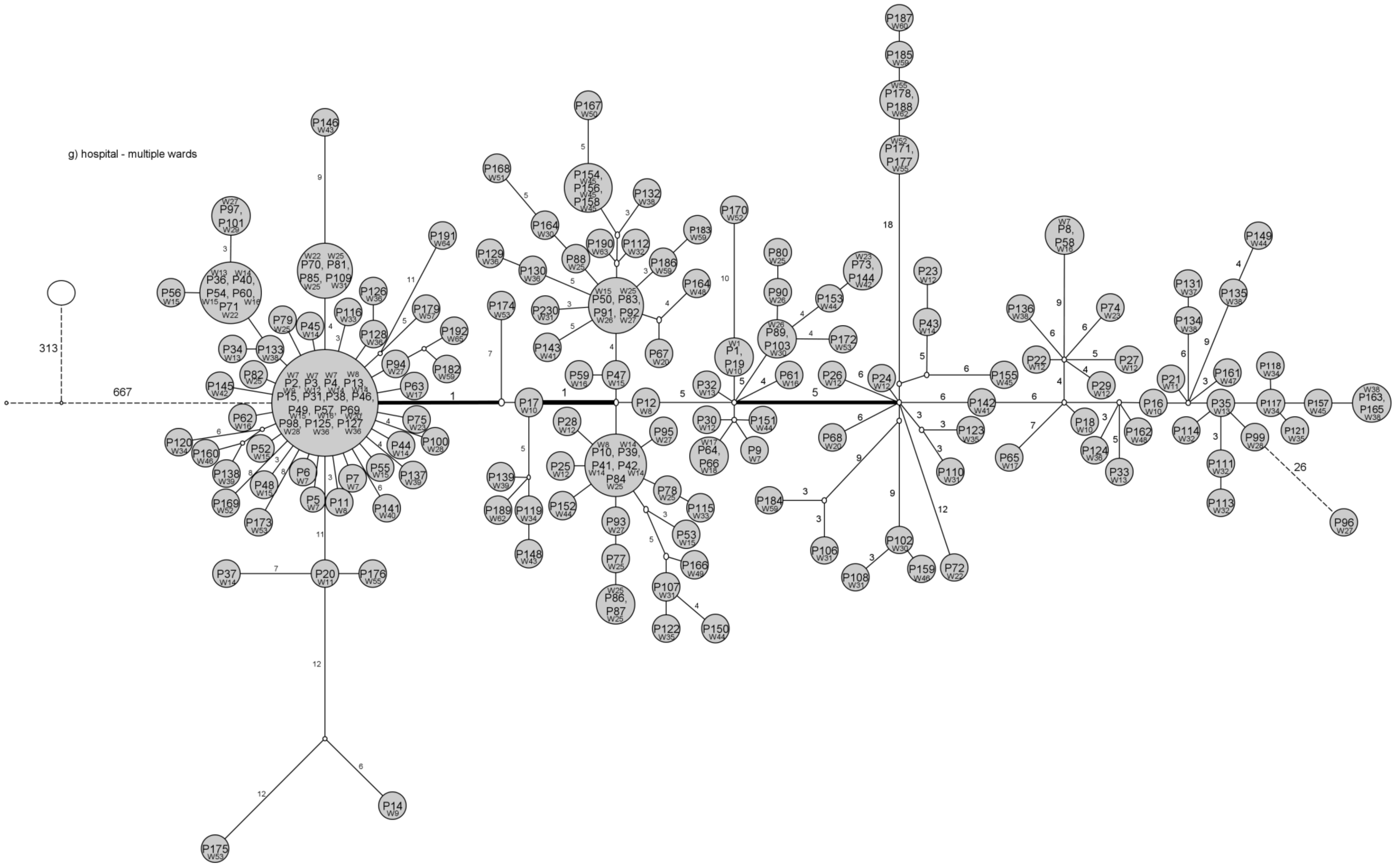
g) hospital - multiple wards
313
667
P146 W43
P97, P101
P36, P40, P54, P60, P71
P56 W15
P70, P81, P85, P109
P2, P3, P4, P13, P15, P31, P38, P46, P49, P57, P69, P98, P125, P127
P20 W11
P37 W14
P176 W55
P14 W9
P175 W53
P17 W10
P174 W53
P167 W50
P154, P156, P158
P50, P83, P91, P92
P10, P39, P41, P42, P84
P86, P87
P12 W8
P170 W52
P1, P19
P89, P103
P73, P144
P187 W60
P185 W59
P178, P188
P171, P177
P8, P58
P142 W41
P16 W10
P35 W13
P117 W34
P157 W45
P163, P165
P96 W27
P64, P66

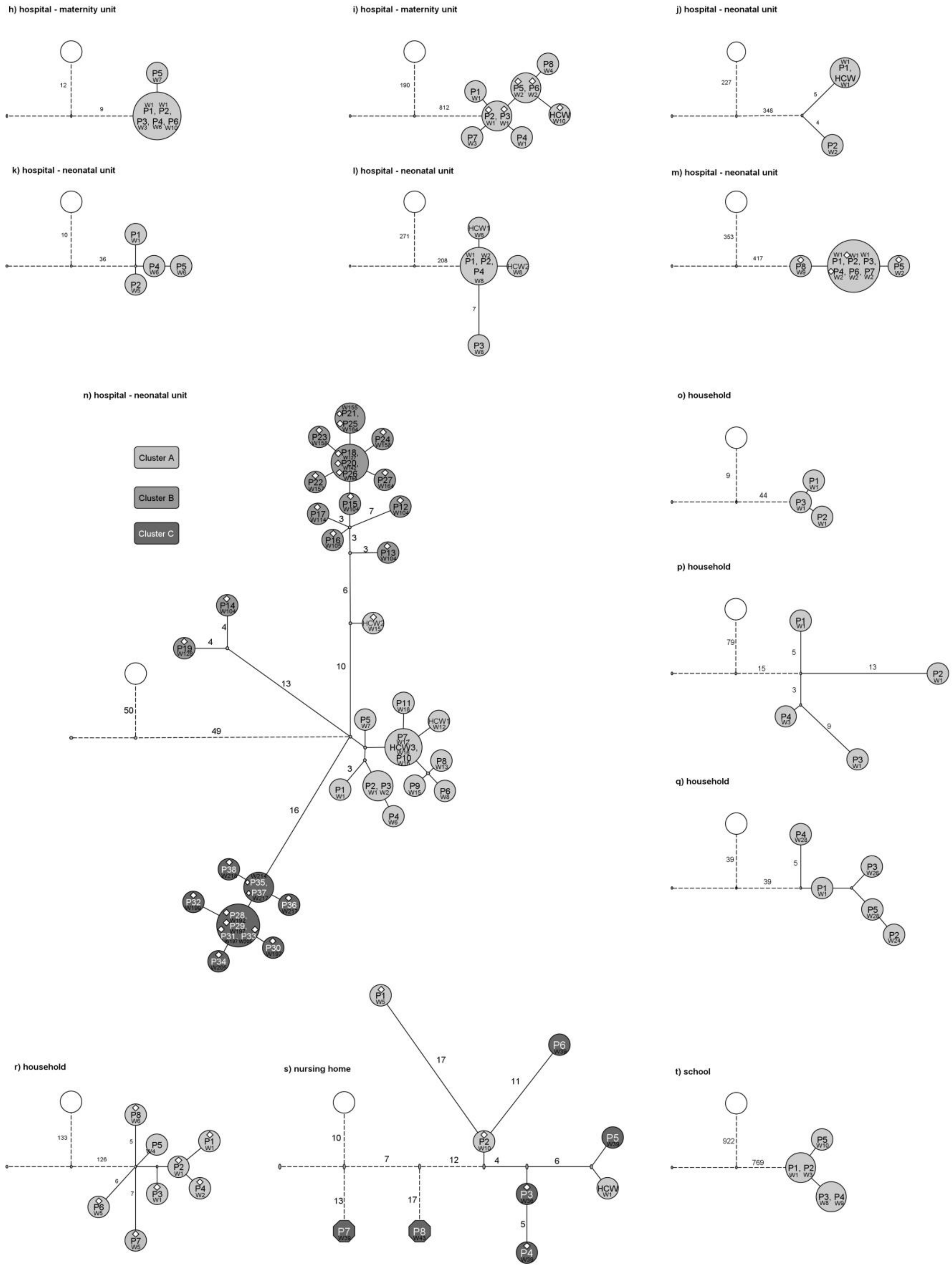

h) hospital - maternity unit
i) hospital - maternity unit
j) hospital - neonatal unit
k) hospital - neonatal unit
l) hospital - neonatal unit
m) hospital - neonatal unit
n) hospital - neonatal unit
Cluster A
Cluster B
Cluster C
o) household
p) household
q) household
r) household
s) nursing home
t) school

5. Effect of mapping
Six outbreaks were remapped to alternative closer reference genomes, either from in-house collections or obtained from Genbank. For the remaining outbreaks, either MRSA 252 was the closest available reference genome, or there was no suitable, closer reference genome available.

Across the 6 outbreaks, mapping to MRSA 252 yielded a total of 66 SNVs, and mapping to the alternative reference yielded 68 SNVs (table 4). The median pairwise difference for the outbreaks was 2.80 for MRSA 252 mapping and 2.77 for alternative reference mapping. In 2 outbreaks, there was no difference in the SNVs identified between standard mapping (to MRSA 252) and within-clonal complex mapping. Standard mapping (to MRSA 252) identified one additional SNV position in 2 outbreaks, while within-clonal complex mapping identified 3 additional SNVs in 1 outbreak, and one additional SNV in the final outbreak. In each case, the increase in SNVs was in the same direction as the increase in percentage of the reference genome covered. There was no effect on overall tree phylogeny for any of the outbreaks.

Table S1: additional SNVs identified by mapping to alternative reference genomes for 6 outbreaks investigated by whole genome sequencing.

Outbreak	CC-match reference genome (Accession no)	% coverage		No of SNVS identified			Mean pairwise difference	
		MRSA 252	CC-match	MRSA 252	CC-match	Difference	MRSA 252	CC-match
b)	MSSA 476 (BX571857.1)	84.0	89.1	4	7	-3	1.97	1.75
c)	EMRSA 15	88.3	86.5	4	4	0	2.17	2.17
h)	EMRSA 15	88.2	86.5	20	20	0	11.70	11.90
i)	USA300	87.6	92.1	24	25	-1	3.43	3.65
j)	EMRSA 15	87.9	86.2	12	11	+1	3.61	3.36
o)	*S. aureus* Newman (AP009351.1)	85.7	84.2	2	1	+1	1.00	0.60
